# A rare cause of cervical swelling: Solitary plexiform neurofibroma

**DOI:** 10.1016/j.amsu.2021.102225

**Published:** 2021-03-16

**Authors:** Ahmed Brahim Ahmedou, Mennouni Mohamed Amine, Oukessou Youssef, Rouadi Sami, Redallah Abada, Roubal Mohamed, Mahtar Mohamed, Regragui Meriem, Karkouri Mehdi

**Affiliations:** a ENT, Head and Neck Surgery Department, Ibn Rochd University Hospital, Faculty of Medicine and Pharmacy, Hassan II, Casablanca, Morocco; b Pathology Department, Centre IBN ROCHD, Casablanca, Morocco

**Keywords:** Solitary neurofibroma, Neck region, Surgery

## Abstract

**Introduction:**

Plexiform cervical neurofibromas are benign neoplasm, extremely rare, difficult to diagnose and to manage. Only some cases have been reported in the literature.

**Case presentation:**

We report the case of a 60-year-old man admitted for a lateral neck mass, for which the surgical indication was the increase in volume of this mass, as well as the aesthetical impairment, the surgical exploration found the tumor attached to the cervical plexus. The excision of the mass was performed without damaging nerve. The pathological study was in favor of a plexiform neurofibroma.

**Discussion:**

Even though Plexiform cervical neurofibroma are extremely rare, and their diagnosis are not often primary evoked in front of any growing mass of this region, the surgeon must keep in mind the existence of these neoplasms as a differential diagnosis of a neck tumor.

**Conclusion:**

Surgery remains the gold standard in the treatment of these locally invasive tumors. It is essential that the surgeon keep in mind the possibility of these tumors as a differential diagnosis of a neck tumor.

## Highlights

• Plexiform cervical neurofibromas are benign neoplasm, extremely rare.
• Difficult to diagnose and to manage.
• Their symptomatology is variable and depends on their topography.
• The clinical aspect is not specific, and the diagnosis is confirmed after a biopsy with anatomopathological examination.
• Surgery remains the gold standard in the treatment of these locally invasive tumors.

## 1 Introduction

We present a case in accordance with SCARE 2020 criteria [1]. Plexiform neurofibroma is a rare, poorly defined benign tumor of the peripheral nerve sheath. They can be single or bilateral, sometimes stepped, superficial or profound. Often considered pathognomonic of neurofibromatosis type 1 (NF1 or von Recklinghausen disease), it can be solitary without NF1.

Formerly known as "plexiform neuroma" or the plexiform neurofibroma is a "royal tumor" and differs from theother types of neurofibroma by the importance of the component schwannian [2]. the diagnosis of plexiform neurofibroma is essentially anatomopathological, particularly without the suggestive context of type I neurofibromatosis. The clinical aspect is not specific, and may evoke a vascular tumor or malformation, a skin or connective tissue tumor, benign or malignant [3]. generally, these tumors are slow-growing. Their symptomatology is variable and depends on their topography [4].

## 2 Case report

A 60-year-old male patient was referred to our otolaryngology department. The patient had an irrelevant past medical history with no known allergies. He had a history of smoking (25 pack-years).he reported with a long standing swelling in the right supraclavicular region. The swelling Persisted for more than ten years.

**Fig 1:**
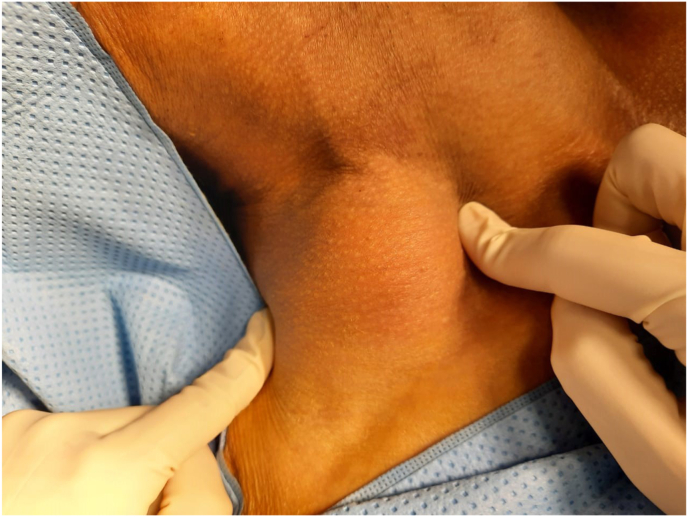
Long standing swelling in the right supraclavicular region.

On clinical examination, it was found that the swelling was firm in nature, non-tender, and non-pulsatile. It was located at the level 5 region and measured 5 × 4 cm, (FIG: 1). No other neck masses. Dermatological, ophthalmological and MRI of the nervous system were normal.

**Fig 2:**
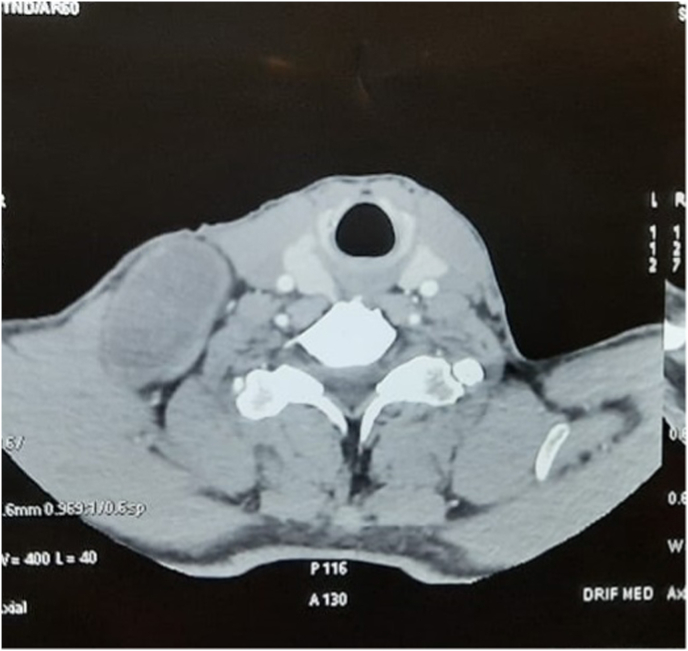
CT scan revealing a large well-defined mass located in the right supraclavicular region.

CT scan revealed a large well-defined mass located in the right supraclavicular region. It measured 7 × 3.5 cm (transverse × anteroposterior measurements). (FIG: 2). Further, MRI scan revealed a low T1 signal with a high T2 signal with heterogeneous enhancement. No other neck masses were found and lymphadenopathy was also absent.

**Fig 3:**
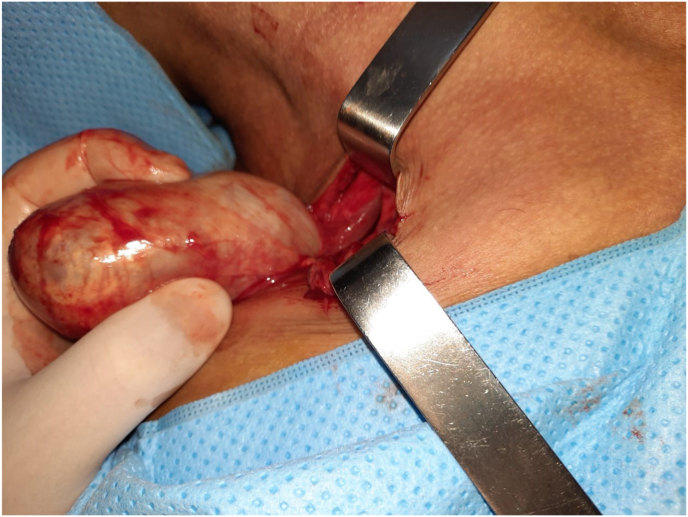
Surgical exploration showing the tumor attached to the cervical plexus.

**Fig 4:**
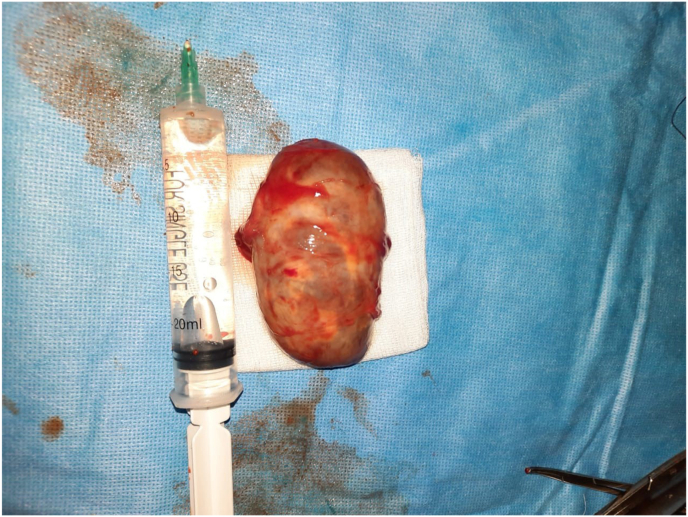
Excision of the mass.

The surgical exploration found the tumor attached to the cervical plexus. (FIG: 3).The excision of the mass was performed without damaging nerve. (FIG: 4).

**Fig 5:**
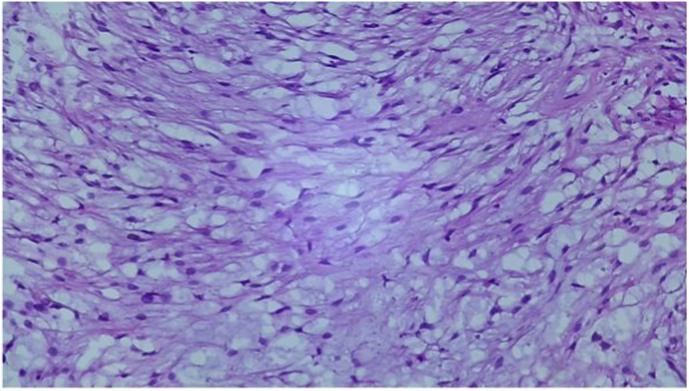
Pathological study.

**Fig 6:**
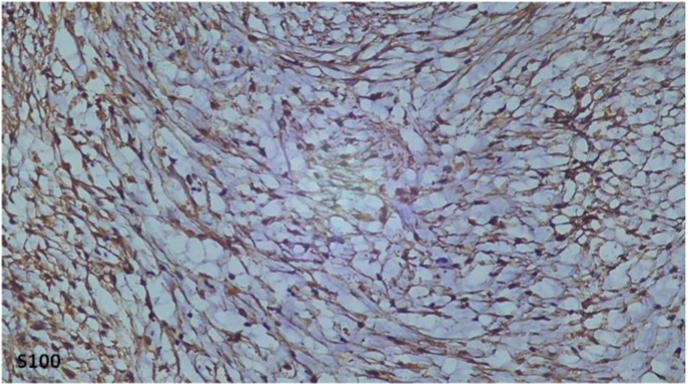
Immunohistochemistry.

In the pathological study, the tumor was poorly limited, unencapsuled, containing numerous neoformed with Cystic tumor proliferation, with elongated spindle-shaped cells with corrugated nuclei, without atypia, on a fibrous background. (FIG: 5).In immunohistochemistry, the Tumor cells express pS100 intensely and diffusely. Tumor cells do not express AML, EMA, or CD99. (FIG: 6). In the light of these findings (clinical evolution, histology, immunohistochemistry (IHC) and the imaging data), the lesion was diagnosed as solitary plexiform neurofibroma.

Regarding the increase in the volume of the mass and the esthetic problem, the patient was planned for a resection churgical. The patient was followed up carefully, without evidence for local recurrence 1 year after surgery.

## 3 Discussion

Neck nerve tumors are rare primary tumors, but have beenknown since 1742 when Haller discovered the carotid corpuscle; then in 1803 Rodier introduced the term “neuroma” for tumors ofthe peripheral nerves [5].

PNF, an uncommon benign tumour, usually presents at birth or in the first years of life [6]. A neck mass is the usual clinical presentation of neck nervetumor. The mass is often isolated and painless although it may bean esthetic blemish, but can also be associated with clinical signsrelated to the function of the affected nerve.Clinical diagnosis is hampered by the numerous differentialpossibilities in this location, but may be suggested by slowprogression, by relative absence of symptoms, and on imaging [7].

Recent advances in immunohistochemistry and imaging allowincreasingly precise diagnosis [8,9]. The majority of such peripheral nerve tumors are benign, but malignant transformation may occur [10].

Plexiform neurofibroma is strongly associated with neurofibromatoses, especially Von's disease Recklinghausen (NF1), where it occurs in 24–32% of the patients [11,12]. The location of the screen-facial display exists within 3–7% of the In the case of a eurofibromatosed type I case [13].

Plexiform neurofibroma belongs to the group of to the 4 types of neurofibromas encountered in the NF1 according to the classification of the consensus conference of 1988 from the National Institute of Health Development [14]:

_ discrete cutaneous neurofibromas of the epidermis or dermis;
_ discrete subcutaneous neurofibromas that are deeper;
_ deep nodular neurofibromas;
_ diffuse plexiform neurofibromas.

These masses can be quite disfiguring, and hemifacial hypertrophy can occur. These tumors are known to cause symptoms ranging from minor discomfort to extreme pain. The consistency of the lesion has been compared to that of a “bag of worms” because of the presence of soft areas interspersed with firm nodular areas. This consistency was well appreciable in the lesion seen in our patient. These lesions sometimes demonstrate a vascular nature.

On CT, the NFPs appear spontaneously isodense with respect to the muscles. They take up contrast only weakly, less than the muscle, which gives them a hypodense aspect (different aspect from schwannomas and neuro-fibrosarcomas) [15]. On MRI, plexiform neurofibromas are typically isointense with muscle on T1 WI, hyperintense on T2 WI and enhance markedly following gadolinium contrast injection [16]. Deep plexiform neurofibromas typically present with a target-like appearance on T2-weighted MR images, with central low-signal intensity and peripheral high-signal intensity. This architecture could accountfor the centrally T2 dark (nerve fibers) and peripherally T2 bright (myxoid) appearance of these target-like lesions. The absence of a target-like appearance does not rule out a lesion being a neurofibroma, particularly if the lesion has a superficial location [15].

At histological analysis, a localized, solitary neurofibroma iscomposed of interlacing fascicles of wavy, elongated cells thatoften contain abundant amounts of collagen. Rarely, myxoid areasand degenerative regions could be found in neurofibromas. Diffuseneurofibroma contains very uniform, prominent fibrillary collagen.Both localized and diffuse neurofibromas are positive for S-100protein at immunohistochemical analysis but this is not a stablefinding [17,18]. It needs to be differentiated from schwannoma which is encapsulated, while plexiform neurofibroma is noncapsulated [19].

In anatomopathology, neurofibroma is poorly limited, not encapsulated, with the absence of thick-walled vessels; Immunohistochemistry is of limited value in this differential diagnosis [20].

Treatment of localized and diffuse neurofibromas (not associ-ated with NF-1) is often surgical resection. Neurofibromas cannotbe easily separated from normal nerve, and complete excision ofthe neoplasm may require sacrifice of the nerve

[17]. While this treatment may be acceptable in superficial lesions, deep lesions can only be managed from the patient's morbidity related to nerve damage and neurological deficits of the surgery [20].

In our case report, total removal of the lesion was performed because it was not relatedto a major cervical nerve. Transformation to malignant peripheral nerve-sheath tumoursis the most feared complication of NF-1 but is less common insolitary neurofiroma and diffuse neurofibroma [21].

The estimatedprevalence of malignant transformation varies from 2 to 29%. Sud-den increase in size of a previously diagnosed neurofibroma wellknown to be clinically and radiologically stable, should be viewedwith great suspicion of malignant transformation and must leadto immediate biopsy. Malignant transformation of these lesions, without association to NF-1, is rare and not well documented in the literature [22].

## 4 Conclusion

Solitary plexiform neurofibroma has to be considered in the differential diagnosis of cervical masses. They are rare tumors with low risk of malignant transformation. If local con-ditions and the general condition of the patient allow surgery, complete resection of the lesion can be proposed.

Surgery remains the gold standard in the treatment of these locally invasive tumors; however, guidelines need to be established regarding the selection of surgical candidates. It is essential that the surgeon keep in mind the possibility of these tumors as a differential diagnosis of a neck tumor.

## Provenance and peer review

Not commissioned, externally peer reviewed.

## Declaration of competing interest

The authors declare having no conflicts of interest for this article

## Registration of research studies

Researchregistry5198.
